# A Novel R2R3-MYB Transcription Factor PqMYB4 Inhibited Anthocyanin Biosynthesis in *Paeonia qiui*

**DOI:** 10.3390/ijms21165878

**Published:** 2020-08-16

**Authors:** Dan Huo, Xiaokun Liu, Yue Zhang, Jingjing Duan, Yanlong Zhang, Jianrang Luo

**Affiliations:** 1 College of Landscape Architecture and Arts, Northwest A&F University, Yangling 712100, China; huodanstu@nwafu.edu.cn (D.H.); liuxiaokun@nwafu.edu.cn (X.L.); zhangyue08@nwafu.edu.cn (Y.Z.); duanjingjing@nwafu.edu.cn (J.D.); zhangyanlong@nwafu.edu.cn (Y.Z.); 2 National Engineering Research Center for Oil Peony, Yangling 712100, China

**Keywords:** *Paeonia qiui*, leaf, anthocyanin, MYB, transcriptional repression

## Abstract

*Paeonia qiui* is a wild tree peony native to China. Its leaves show a clear purple-red color from the germination to the flowering stage, and it has high leaf-viewing value. A MYB transcription factor gene, designated as *PqMYB4*, was isolated from leaves of *P. qiui* based on transcriptome datas. The full-length cDNA of *PqMYB4* was 693 bp, encoding 230 amino acids. Sequence alignment and phylogenetic analysis revealed that PqMYB4 was a R2R3-MYB transcription factor clustered with AtMYB4 in *Arabidopsis thaliana*. Moreover, it contained a C1 motif, an EAR repression motif and a TLLLFR motif in the C-terminal domains, which were unique in transcription repression MYB. Subcellular location analysis showed that PqMYB4 was located in the cell nucleus. *PqMYB4* was highly expressed in the late stage of leaf development, and was negatively correlated with the anthocyanin content. The petiole of wild-type Arabidopsis seedlings was deeper in color than the transgenic lines of *PqMYB4* and showed a little purple-red color. The seed coat color of Arabidopsis seeds that overexpressed *PqMYB4* gene was significantly lighter than that of wild-type seeds. In transgenic Arabidopsis, the expression level of *AtCHS*, *AtCHI*, *AtDFR* and *AtANS* were down-regulated significantly. These results showed that PqMYB4 was involved in the negative regulation of anthocyanin biosynthesis in tree peony leaves, which can control the anthocyanin pathway genes. Together, these findings provide a valuable resource with which to further study the regulatory mechanism of anthocyanin biosynthesis in the leaf of *P. qiui*. They also benefit the molecular breeding of tree peony cultivars with colored leaf.

## 1. Introduction

Anthocyanins are a class of flavonoids derived from phenylalanine. They are water-soluble pigment that can make plants exhibit colors ranging from orange-red to blue-purple. Anthocyanins are synthesized in the cytosol and transported to the vacuole for storage. In plants, anthocyanins are often present in pollen, petals and fruits, attracting pollinators and spreading seeds; moreover, they have a protective effect against a series of biological or abiotic stress, such as pathogen infection, strong light, low temperature and phosphate stress [1]. At present, the anthocyanin biosynthetic pathway is well understood. The enzymes required in this pathway mainly include chalcone synthase (CHS), chalcone isomerase (CHI), flavanone 3-hydroxylase (F3H), flavonoid 3′-hydroxylase (F3′H), flavonoid 3′,5′-hydroxylase (F3′5′H), dihydroflavonol-4-reductase (DFR) and anthocyanidin synthase (ANS) [2]. CHS is the key enzyme in the early stage of anthocyanin biosynthesis, and DFR and ANS are key enzymes in the late stage of anthocyanin synthesis, both of which play an important role in the accumulation of anthocyanins [3,4]. In addition, anthocyanin biosynthesis is also regulated by transcription factors that can recognize specific DNA motifs in the promoter of structural genes [5].

MYB transcription factor is one of the major transcription factors regulating anthocyanin synthesis [6]. R2R3-MYBs are the most extensive transcription factors in plants [7]. They contain R2 and R3 conserved domains at the N-terminus, and usually contain transcriptional activation or repression motifs at the C-terminus [8]. Many R2R3-MYBs transcription factors that promote anthocyanin synthesis have been identified in different plants, such as tobacco (*Nicotiana tabacum*) [9], *Arabidopsis thaliana* [10], grapevine (*Vitis vinifera*) [11], apple (*Malus domestica*) [12] and pear (*Pyrus pyrifolia*) [13].

In addition to MYB activators, two types of MYB repressors, R3-MYBs and R2R3-MYBs, have also been identified. R3-MYBs contain a single MYB DNA binding domain, such as Arabidopsis CAPRICE (CPC), TRIPTYCHON (TRY) and MYBL2 [14,15], and petunia (*Petunia hybrida*) MYBx [16]. Most of CPC-like R3-MYBs were negatively associated with anthocyanin accumulation, root hair and trichome development [17,18]. The first R2R3-MYB transcriptional repressor was AmMYB308 in snapdragon (*Antirrhinum majus*) [19]. Subsequently, R2R3-MYB inhibitors were also discovered in other species, including FaMYB1 in strawberry (*Fragaria × ananassa*) [20], PhMYB27 in petunia [21], VvMYBC2-L1/3 and VvMYB4-like in grapevine [22,23]. All of them belong to subgroup 4 R2R3 type MYB transcription factors. In petunia, overexpression of *PhMYB27* gene can significantly reduce the accumulation of anthocyanins in petals and leaves. In addition, a decrease in the accumulation of proanthocyanidins can also be observed in seeds. The gene silencing system of *PhMYB27* can increase the accumulation of anthocyanins [21]. In grapevine, *VvMYBC2-L1* and *VvMYBC2-L3* were highly expressed in pulp, when they were overexpressed in petunia, the accumulation of anthocyanins in the petals decreased, and proanthocyanidin content was also reduced when they were overexpressed in the root hair of grapevine [22].

*Paeonia qiui* is a wild tree peony native to China. The leaves show a clear purple-red color from the germination to the flowering stage [24], which has high leaf-viewing value. At present, there is no report on the MYB transcription factor that negatively regulates anthocyanin synthesis in tree peony. In this study, based on the transcriptome sequencing, an anthocyanin biosynthesis-related R2R3-MYB repression gene, *PqMYB4*, was isolated, and its function in inhibiting anthocyanin synthesis was investigated in transgenic Arabidopsis plants. This study provided a basis for revealing the mechanism of leaf color change in *P. qiui*, and provided genetic resources for the molecular breeding of the tree peony cultivars with colored leaf.

## 2. Results

### 2.1. Characterization of PqMYB4

**Figure 1:**
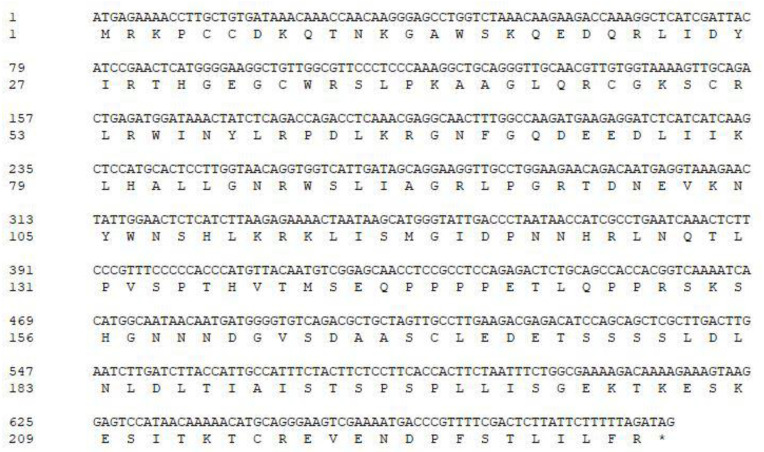
Nucleotide sequence and deduced amino acid sequence of *PqMYB4*. The deduced amino acid sequence was shown underneath the corresponding nucleotide sequence, and stop code was indicated with *.

**Figure 2:**
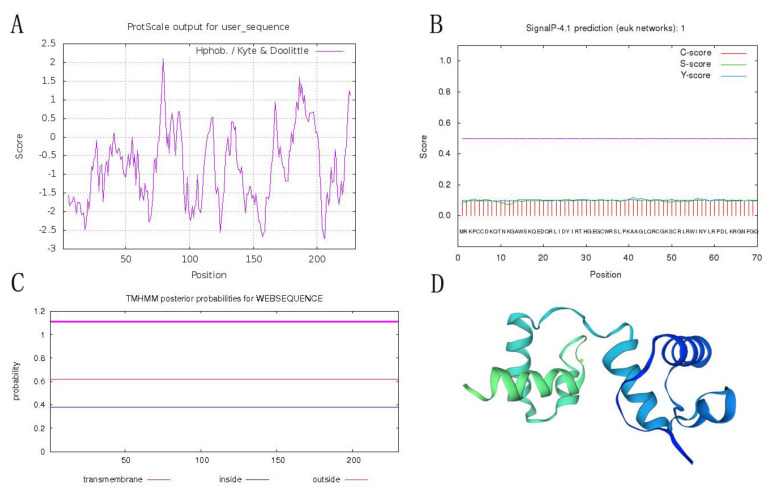
Bioinformatics analysis of PqMYB4 protein. (**A**) hydrophilic and hydrophobic analysis; (**B**) signal peptide analysis; (**C**) transmembrane analysis; (**D**) tertiary structure prediction.

The open reading frame (ORF) of *PqMYB4* was 693 bp, which encodes a protein of 230 amino acids residues in length (Figure 1). The online software ProtParam analysis showed that the theoretical molecular weight of PqMYB4 was about 25.93 kD; the theoretical isoelectric point was 8.83. The protein instability coefficient was 55.91, indicating that it was an unstable protein. The hydrophilic mean (GRAVY) was −0.783, indicating that the protein was a hydrophilic protein (Figure 2A). SignalP 4.1 Server and TMHMM predicted that PqMYB4 did not have a signal peptide site and transmembrane region, indicating that it belonged to one of non-secreted proteins and non-transmembrane proteins (Figure 2B,C). Secondary structure prediction showed that PqMYB4 contained 30.00% α-helix, 6.96% β-turn, 12.61% extended chain and 50.43% random coil. The three-dimensional structure was constructed by SWISS-MODEL, the similarity of the model was 65.09% with the transcription factor WER (Figure 2D). Conservative domain analysis revealed that there were two conserved domains (repeat R) at the N-terminus. The amino acid sequence at positions 11–61 was the R2 conserved domain, and the amino acid sequence at positions 64–112 was the R3 conserved domain, indicating that PqMYB4 belonged to the R2R3-MYB subfamily (Figure 1).

### 2.2. Phylogenetic Analysis and Sequence Alignment of PqMYB4

**Figure 3:**
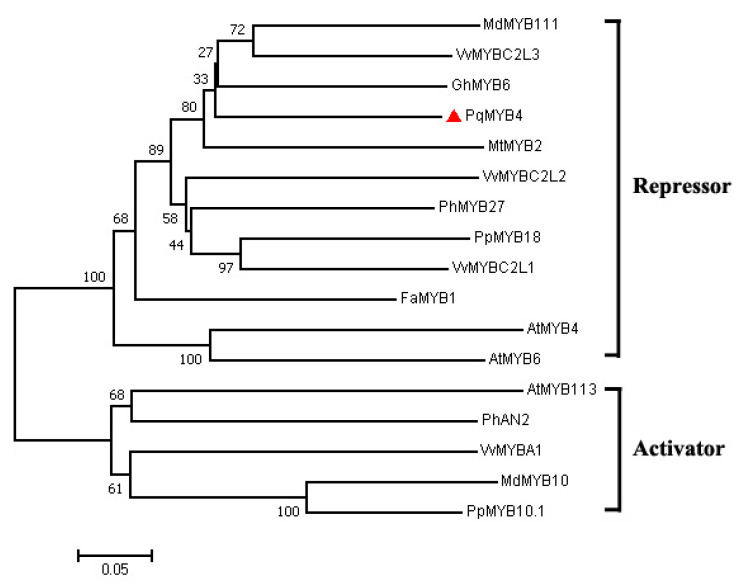
Phylogenetic analysis of PqMYB4 and the MYB proteins known to regulate anthocyanin biosynthesis from other species. PqMYB4 is highlighted with a red triangle. The neighbor-joining method with MEGA software was used to construct the phylogenic tree. Bootstrap values as a percentage of 1000 replicates are indicated at corresponding branch nodes. Scale bar represents the number of amino acid substitutions per site. The GenBank accession numbers are below: *Malus domestica*, MdMYB111 (ADL36754), MdMYB10 (ABB84754), *Vitis vinifera*, VvMYBC2-L1 (ABW34394), VvMYBC2-L2 (ACX50288), VvMYBC2-L3 (AIP98385), VvMYBA1 (BAD18977), *Gossypium hirsutum*, GhMYB6 (AAC04720), *Medicago truncatula*, MtMYB2 (XP_003616388), *Petunia hybrida*, PhMYB27 (AHX24372), PhAN2 (AAF66727), *Prunus persica*, PpMYB18 (ALO81021), PpMYB10.1 (XP_007216530), *Fragaria × ananassa*, FaMYB1 (AAK84064), *Arabidopsis thaliana*, AtMYB4 (AAC83582), AtMYB6 (Q38851), AtMYB113 (NP_176811).

Phylogenetic analysis indicated that PqMYB4 was clustered with other inhibitory transcription factors, such as cotton GhMYB6 [25], grapevine VvMYBC2L3 [22], apple MdMYB111 and *Medicago truncatula* MtMYB (Figure 3) [26,27]. The results suggested that PqMYB4 may have a similar function to other known MYB transcriptional repressors.

**Figure 4:**
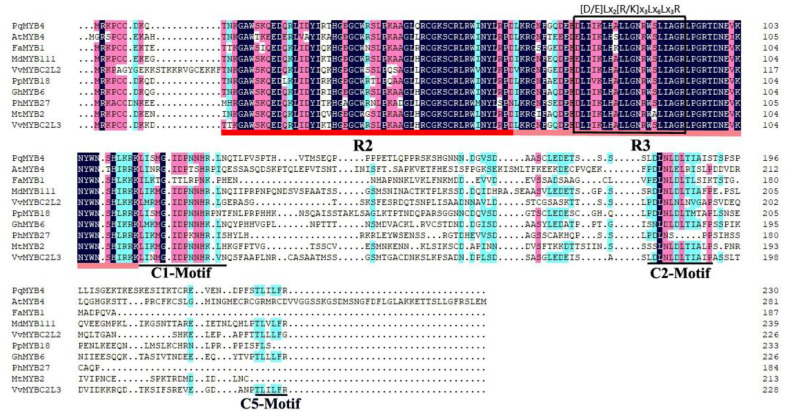
Alignment of PqMYB4 deduced amino acid sequence with similar proteins from other species. Alignment was conducted using DNAMAN Version 8. The R2 and R3 MYB domains shown refer to two repeats of the MYB DNA-binding domain of MYB proteins. The black box shows the [D/E]Lx_2_[R/K]x_3_Lx_6_Lx_3_R motif, and black line shows the C1, C2, C5 motif.

The deduced amino acid sequence of PqMYB4 was aligned with similar MYB proteins from other species using DNAMAN software version 8. The results showed that the protein of PqMYB4 shared the highest identity of 54.24% with grapevine VvMYBC2L3, the following were cotton GhMYB6, apple MdMYB111 and peach PpMYB18 [28], which were 50.51%, 50.51% and 47.12%, respectively (Figure 4). The PqMYB4 showed the lowest identity with strawberry FaMYB1 [29], which was 38.64%. A sequence analysis revealed that PqMYB4 contained the R2 and R3 MYB DNA-binding domains in N-terminus, indicating that PqMYB4 was a R2R3-MYB transcription factor (Figure 4). PqMYB4 contained a motif, [D/E]Lx_2_[R/K]x_3_Lx_6_Lx_3_R, which is important for interaction with a basic helix-loop-helix (bHLH) protein (Figure 4) [30]. Furthermore, a C1 motif (LIsrGIDPxT/SHRxI/L), a C2 motif (pdLNL^D^/_E_Lxi^G^/_S_) and a C5 motif (TLLLFR) were found in the C-terminus of PqMYB4, which are suggested to be involved in transcriptional repression (Figure 4) [31]. Thus, PqMYB4 may be involved in transcriptional repression, which was consistent with the conclusion of the polygenetic analysis (Figure 3).

### 2.3. Subcellular Localization of PqMYB4 Protein

**Figure 5:**
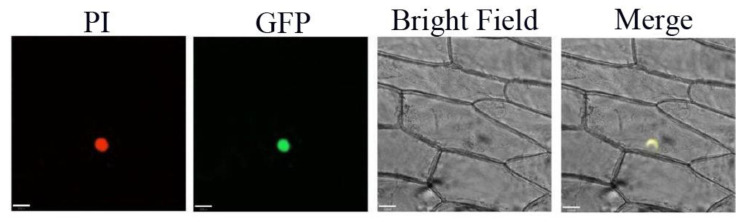
Subcellular location of GFP fusion of PqMYB4. Bars, 33 µm.

To investigate the subcellular localization of PqMYB4, the *PqMYB4* coding sequence without stop codon was fused to the 5′ terminus of gene coding green fluorescent protein (GFP), then, the plasmid pCAMBIA1301-*PqMYB4*-GFP was transformed into onion cells. Onion cells expressing the *PqMYB4*-GFP fusion protein showed a strong signal in the nucleus, the result showed that PqMYB4 was located in the cell nucleus (Figure 5).

### 2.4. PqMYB4 Expression Negatively Correlates with Anthocyanin Biosynthetic Gene Expression and Anthocyanin Accumulation in P. qiui

**Figure 6:**
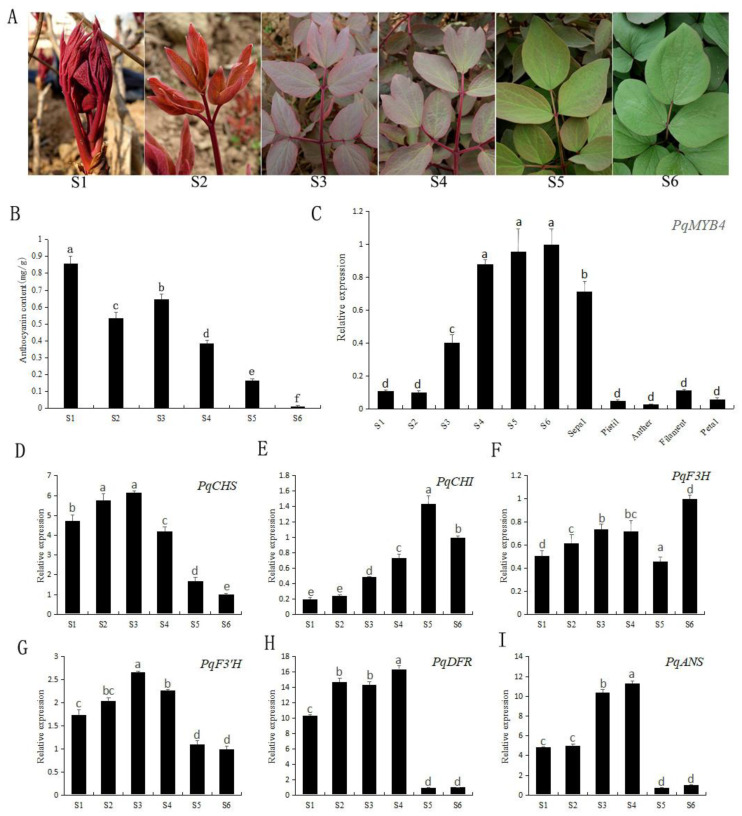
Anthocyanin content, expression levels of *PqMYB4* and anthocyanin biosynthetic genes in different leaf color stages in *P. qiui*. (**A**) the leaf phenotype of *P. qiui* in different leaf color stages. S1: germination stage with red color, S2: sprout leaves stage with light red color, S3: red stage, S4: red with green stage, S5: green with red stage, S6: green stage; (**B**) anthocyanin content; (**C**) the expression level of *PqMYB4*; (**D**–**I**) the expression level of anthocyanin-related biosynthetic genes, *PqCHS* (**D**), *PqCHI* (**E**), *PqF3H* (**F**), *PqF3’H* (**G**), *PqDFR* (**H**), *PqANS* (**I**). a, b, c, d, e and f indicate significant difference at *p* ≤ 0.05 level by Duncan test.

The expression levels of *PqMYB4* and anthocyanin biosynthetic genes in different leaf stages were revealed by qRT-PCR. The anthocyanin content was measured with a UV spectrophotometer (Figure 6). The results showed that the anthocyanin content in leaves at S1 was the highest, then decreased, and the anthocyanin content at S6 was the lowest. However, the anthocyanin content increased slightly in the S3 period, which was basically consistent with the phenotypic change of the leaves in *P. qiui*. (Figure 6A,B). As for the expression level of *PqMYB4* (Figure 6C), the results showed that, with the growth and development of *P. qiui* leaves, the expression level of *PqMYB4* gradually increased; the first two stages with the minimum value of transcript level and peaked at S6. The expression level of *PqMYB4* in the green leaf stage is about 10 times than that in the red leaf stage. In general, the trend of *PqMYB4* expression level was negatively correlated with that of anthocyanin content. Additionally, *PqMYB4* had a high expression level in sepals, followed by filaments and petals, and had a low expression level in pistils and anthers.

In addition, the expression levels of anthocyanin biosynthetic genes were also analyzed. The result showed that *PqCHS*, *PqF3′H*, *PqDFR* and *PqANS* genes presented a basically consistent trend, which increased first, and then decreased (Figure 6D–I). The expression levels of these genes were higher at the early stages (S1–S4) and lower at the late stages (S5, S6). That is to say, the expression trend of these genes was positively correlated with the trend of anthocyanin content, and negatively correlated with the trend of *PqMYB4*.

### 2.5. PqMYB4 Was Not a Transcriptional Activator

**Figure 7:**
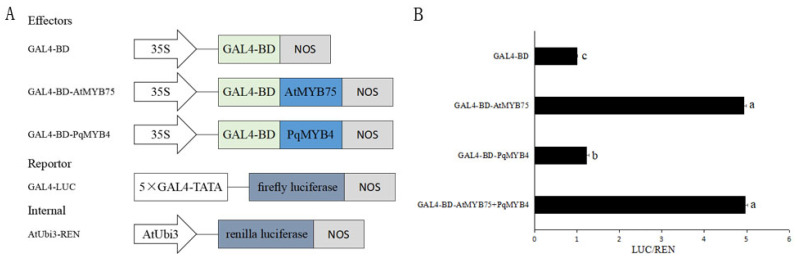
Dual Luciferase Transient Transfection Assay of PqMYB4. (**A**) scheme of the constructs used in the Arabidopsis protoplast cotransfection assay; (**B**) the relative luciferase activity (LUC/REN). a, b and c indicate significant difference at *p* ≤ 0.05 level by Duncan test.

To examine the transcriptional activity of PqMYB4, we used transient reporter assays (Figure 7). Effector plasmids containing *AtMYB75*, which is a transcriptional activator [7] or *PqMYB4*, reporter plasmid containing firefly luciferase (LUC) and internal plasmid containing renilla luciferase (REN) were delivered into Arabidopsis protoplast by PEG-mediated transformation of protoplasts (Figure 7A). As shown in Figure 7, an increase of more than fourfold in relative luciferase activity was induced by the expression of *AtMYB75*, compared with that induced by the GAL4-BD control (Figure 7B). PqMYB4 did not induce the increase in relative luciferase activity (Figure 7B), suggesting that PqMYB4 was not a transcriptional activator.

### 2.6. PqMYB4 Suppressed Anthocyanin Accumulation and the Expression of Anthocyanin Pathway Genes

**Figure 8:**
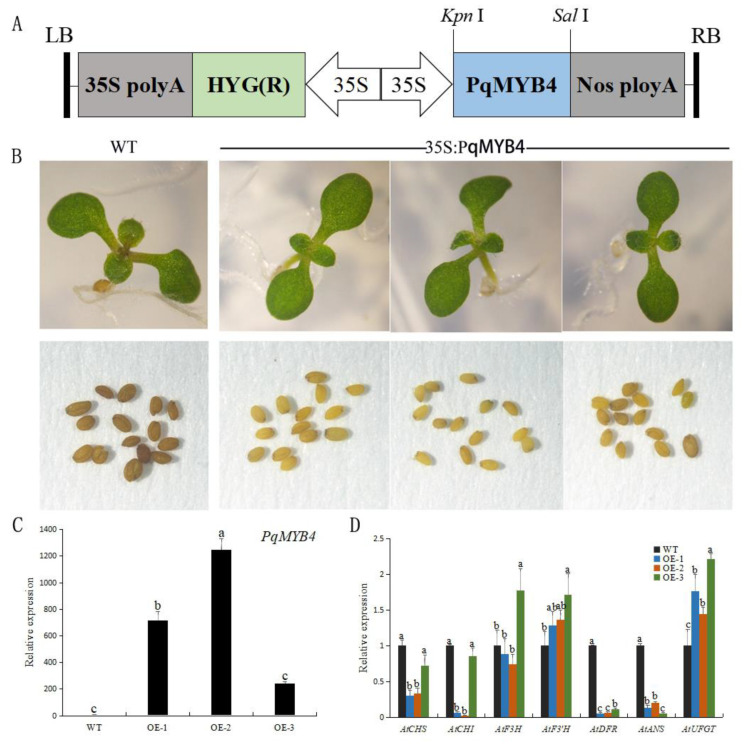
Diagrammatic presentation of vector construct, phenotype and the effect of *PqMYB4* overexpression in transgenic Arabidopsis plants. (**A**) diagrammatic presentation of vector construct; (**B**) phenotype; (**C**) the expression level of *PqMYB4* gene; (**D**) the expression level of anthocyanin-related structure genes, *AtCHS*, *AtCHI*, *AtF3H*, *AtF3′H*, *AtDFR*, *AtANS*, *AtUFGT*. a, b and c indicate significant differences at *p* ≤ 0.05 level by Duncan test.

To characterize the function of PqMYB4, overexpression of *PqMYB4* in Arabidopsis was carried out. The results showed that the petiole of wild-type (WT) Arabidopsis seedlings grown in the medium containing sucrose were deeper in color than the transgenic lines of *PqMYB4*, and showed a little purple-red color (Figure 8B). In addition, the seed coat color of Arabidopsis overexpressing *PqMYB4* was significantly lighter than that of wild type (Figure 8B).

Additionally, the expression levels of anthocyanin biosynthesis-related genes (*AtCHS*, *AtCHI*, *AtF3H*, *AtF3’H*, *AtDFR*, *AtANS* and *AtUFGT*) and *PqMYB4* in the wild-type and the transgenic Arabidopsis plants were analyzed by qRT-PCR assay. Compared to wild-type, *PqMYB4* gene was highly expressed in the transgenic plants, and there was little or no expression in wild-type Arabidopsis (Figure 8C). The expression levels of *AtDFR* and *AtANS* were all significantly down-regulated in three transgenic lines, while the expression levels of *AtCHS* and *AtCHI* genes were obviously down-regulated in two transgenic lines (OE-1, OE-2). The expression levels of *AtF3’H* and *AtUFGT* genes were up-regulated compared to wild type (Figure 8D).

## 3. Discussion

*P. qiui* is a typical spring color leaf ornamental plant. With the growth and development, its leaf color shows a transition from purple-red to green. In this study, the anthocyanin content of leaves in different periods showed a gradual decreasing trend, which was consistent with the leaf phenotype and the expression levels of *PqCHS*, *PqF3’H*, *PqDFR* and *PqANS* genes (Figure 6). In the red leaf stages, the anthocyanin content was higher, and the expression levels of *PqCHS*, *PqF3’H*, *PqDFR* and *PqANS* were up-regulated, while in the green leaf stages, the anthocyanin content was lower, and the expression levels of *PqCHS*, *PqF3’H*, *PqDFR* and *PqANS* were down-regulated. The expression of *PqMYB4* in different tissues showed that it was high expression in the green leaf stages, and predominantly expressed in green tissues, low expression in other tissues (Figure 6C). It indicated that the expression level of *PqMYB4* was basically opposite to the trend of anthocyanin content. *MdMYB16* was a transcription repressor gene found in apple, which was expressed higher in white pulp and lower in red pulp [32]. VvMYBC2L2 was a transcription repression MYB in grapevine. It had the highest expression in the green skin of the early stage of grapevine (*Vitis vinifera* ‘Yatomi Rose’), and had the lowest expression in the red skin of the mature stage [33], which was consistent with the expression pattern of *PqMYB4* in this study. However, *PpMYB18* was a transcription repression gene found in peach, its expression pattern was different from the transcription repression genes above-mentioned. *PpMYB18* gene was highly expressed in fruit at ripening and/or juvenile stages when anthocyanins or PAs were being synthesized [28]. These showed that the expression patterns of transcription repressors in different plants were not completely consistent, which has a certain diversity.

In our dual luciferase transient transfection assay, GAL4-BD-PqMYB4 did not increase the relative Luc activity compared with that of the GAL4-BD control (Figure 7B). It indicated that PqMYB4 was not a transcriptional activator. GAL4-BD-PqMYB4 did not reduced the Luc activity (Figure 7B), but it still may be a transcriptional repressor. PqMYB4 contained a motif, [D/E]Lx_2_[R/K]x_3_Lx_6_Lx_3_R, which is important for interaction with a basic helix-loop-helix (bHLH) protein (Figure 4) [30]. The motif [D/E]Lx_2_[R/K]x_3_Lx_6_Lx_3_R is very important for the inhibition function of MYB transcriptional repressor, it means that these MYB may be need bHLH to play its inhibition function [34]. Therefore, in transient transfection assay, the repression activity of PqMYB4 may also need bHLH. It needs further study.

Overexpression of *VvMYBC2L2* in tobacco plants inhibited the accumulation of anthocyanins in corolla. Expression analysis showed that the expression of flavonoid-related genes *NtCHS*, *NtDFR*, *NtLAR* and *NtUFGT* in tobacco were significantly reduced [22,33]. The ectopic expression of the MYB transcription suppressor gene *GbMYBF2* in Arabidopsis could inhibit the synthesis of anthocyanins and down-regulate the expression of structural genes *CHS*, *F3H*, *FLS* and *ANS* [35]. The overexpression of *PhMYB27* gene significantly reduced the expression of *F3′5′H*, *DFR*, *ANS*, *3GT*, *5GT* and *GST* genes compared to the control plants [21]. In Chinese narcissus (*Narcissus tazetta* var. *chinensis*), when *NtMYB5* was overexpressed in tobacco, the flower color became lighter, and the expression levels of most structural genes in the anthocyanin biosynthesis pathway were reduced [36]. In this study, no anthocyanin accumulation was observed in *PqMYB4* transgenic Arabidopsis seedlings, while a little accumulation of anthocyanins was observed in wild type (Figure 8B). Real-time quantitative PCR results showed that expression level of anthocyanin synthesis structural genes *AtCHS*, *AtCHI*, *AtDFR*, *AtANS* in transgenic plants were significantly down-regulated, especially *AtDFR* and *AtANS* (Figure 8D). In addition, the seed coat color of *A. thaliana* overexpressing *PqMYB4* gene was significantly lighter than that of wild type (Figure 8B). All of these showed that PqMYB4 had the function of inhibiting the synthesis of flavonoids (anthocyanins).

Previous research found that there was a C1 motif, a C2 motif (EAR motif), a C3 motif (CX1-2CX7-12CX2C) and a C4 motif (FLGLx4-7V/LLD/GF/YR/Sx1LEMK) at the C-terminus in transcriptional repression factors; some also had a TLLLFR motif [15,37]. Substitution or deletion of the C1 motif may reduce the inhibitory activity of PpMYB18 [28]. However, FaMYB1 lacking the C1 motif could still inhibit the synthesis of flavonoids [20,29]. The C1 motif was also found in the AtMYB5 homologs which have an activation role and belong to the MAV clade [38]. EAR conserved motif existed in most of the MYB proteins with inhibitory effect, while C3 or C4 conserved motif mainly existed in AtMYB4-like transcription inhibitors [31]. All of the members of Arabidopsis R2R3-MYBs in subgroup 4 have ethylene-responsive factor related repression motif EAR, such as AtMYB3, AtMYB7, AtMYB4 and AtMYB32, which can participate in transcriptional repression [39]. The core sites of proteins containing EAR motif in Arabidopsis showed a conservative pattern of DLNxxP or LxLxL sequences [40]. Mutation of the EAR motif of PtMYB182 did not result in a detectable reduction of repressor function [34]. The over-expression of apple *MdMYB32* gene in red apple callus reduced the accumulation of anthocyanins, but the content of anthocyanins didn’t change after the EAR motif was mutated [41]. The TLLLFR motif was first discovered in the R3-MYBs flavonoid inhibitor AtMYBL2, it was also present in some FaMYB1-like transcription inhibitors, such as VvMYBC2, VvMYB4-like and PtMYB182 [34]. In Arabidopsis, TLLLFR appeared to be a repression motif [15], but in poplar, disruption of the TLLLFR motif in PtMYB182 did not inhibit the repressor activity [34]. In addition, it has been found that the bHLH binding motif in the R3 domain is necessary for the passive R2R3-MYB inhibitory factors [28,34]. These results suggest that the repressive activity of MYB repressors may be controlled by different mechanisms in different plants. In this study, a R2R3-MYB transcription factor, PqMYB4 was isolated, which had typical R2 and R3 conserved domains. The R3 domain had a [D/E]Lx_2_[R/K]x_3_Lx_6_Lx_3_R motif that could interact with bHLH protein, and it had a C1 motif, an EAR motif, and contained a TLLLFR motif. However, which motif that determines the inhibitory function of PqMYB4 is requires further research.

## 4. Materials and Methods

### 4.1. Plant Materials

*P. qiui* plants were grown under field conditions in Northwest A&F University, Yangling Shaanxi, China. The samples were collected in the morning during March and April in 2018, including sepals, petals, pistils, stamens and the leaves at six different leaf color stages (S1, S2, S3, S4, S5, S6) (Figure 6A). In order to avoid the effect of other organisms (arthropods, bacteria, fungi, etc.) on gene expression, only healthy, undiseased and non pest injured leaves were collected. All samples were collected from the same plant and washed three times with distilled water, and immediately frozen in liquid nitrogen, then stored at −80 °C for RNA extraction and pigments analysis.

The seeds of Arabidopsis were germinated in a growth chamber at 26/22 °C day/night temperature, with a 70% relative humidity and light/dark cycle of 15/9 h. Arabidopsis ecotype Columbia (Col) was used for ectopic overexpression experiment.

### 4.2. Total RNA Extraction and cDNA Synthesis

The total RNA of tissue and leaf samples was isolated using the TIANGEN RNA Prep Pure Plant kit, according to the manufacturer’s instructions (Tiangen, Beijing, China). The quality and concentration of RNA samples were tested by Goldview-stained agarose gel electrophoresis and spectrophotometric analysis, respectively. The first-strand cDNA was synthesized from 1 μg of DNA-free RNA samples using a PrimeScript^®^ RT reagent Kit with gDNA Eraser (Takara, Otsu, Shiga, Japan), according to the manufacturer’s instructions. First, the reaction mixture contained 2 μL of 5× gDNA Eraser Buffer, 1 μL of gDNA Eraser and corresponding volume RNA samples, ddH_2_O added up to 10 μL, 42 °C 2 min to remove genomic DNA; Next, reverse transcription reaction: add 1 μL of PrimeScript RT Enzyme Mix I, 1 μL of RT Primer Mix, 4 μL of 5× PrimeScript Buffer 2 and 4 μL of RNase-Free ddH_2_O to the first reaction solution, total reaction volume was 20 μL. The PCR program was carried out with an initial step of 37 °C for 15 min; then 85 °C for 5 s and 4 °C for storage.

### 4.3. Full-Length cDNA Clone of PqMYB4

Based on previous transcriptomic datas of tree peony leaves, a MYB transcription factor gene related to leaf anthocyanin biosynthesis, *PqMYB4*, was screened. According to the transcriptome sequence of *PqMYB4*, gene specific primers PqMYB4-F and PqMYB4-R were designed by Oligo7.0 software (Table 1), and the full-length gene amplification was carried out using the cDNA of *P. qiui* leaves as a template. The PCR program was carried out by an initial step of denaturing the cDNA at 94 °C for 10 min; then followed by 40 cycles of 94 °C for 30 s, 55 °C for 30 s, 72 °C for 60 s, and by a final extension of 72 °C for 10 min. After detecting the PCR products by agarose gel electrophoresis, the specific bands of the expected size PCR products were recycled by Agarose Gel DNA Extraction Kit (Takara, Otsu, Shiga, Japan). Then, the expected products were ligated into the pMD19-T vector (Takara, Otsu, Shiga, Japan), placed at 4 °C overnight, and transformed the ligation products into *Escherichia coli* competent cell. After positive screening and colony PCR identification, the correct bacterial solution was sent to the company for sequencing.

### 4.4. Bioinformatics Analysis of PqMYB4

Open reading frame and deduced amino acid sequence of PqMYB4 was searched by ORF Finder online tool in NCBI (https://www.ncbi.nlm.nih.gov/orffinder/). The amino acid composition, protein molecular weight, theoretical isoelectric point (pI) and stability were predicted by using online software ProtParam (http://web.expasy.org/protparam/). The hydrophobic property and charge distribution were analyzed by using online software ProtScale (https://web.expasy.org/protscale/). The transmembrane domain of PqMYB4 protein was analyzed by using TMHMM server 2.0 software (http://www.cbs.dtu.dk/services/TMHMM). The signal peptide of PqMYB4 was predicted by SignalP 4.1 Server (http://www.cbs.dtu.dk/services/SignalP/). The secondary and tertiary protein structure predictions were conducted by using SOPMA (https://npsa-prabi.ibcp.fr/cgi-bin/npsa_automat.pl?page=/NPSA/npsa_sopma.html) and SWISS MODEL (https://swissmodel.expasy.org/), respectively. The conserved domain was identified by SMART (http://smart.embl-heidelberg.de/smart/set_mode.cgi?NORMAL=1). The homologous sequences alignment of PqMYB4 with other species was performed by DNAMAN version 8 software, and the phylogenetic tree was constructed using Neighbor-Joining method (NJ) of MEGA7.0 software.

### 4.5. Subcellular Localization

For subcellular localization analysis, the *PqMYB4* ORF region without the stop codon was inserted into the binary vector pCAMBIA1301-GFP, digested with *BamH* I/*Sal* I restriction sites to generate 35S::*PqMYB4*-GFP fusion construct. This constructed plasmid was bombarded into onion epidermal cell using a Biolistic PDS1000-instrument (Bio-Rad, CA, USA). After incubation at 25 °C for at least 16 h in the dark, samples were observed under a confocal laser scanning microscope.

### 4.6. Dual Luciferase Transient Transfection Assay

The p35S-GAL4-BD, the effector plasmid, the reporter plasmid containing firefly luciferase and internal plasmids containing Renilla luciferase, respectively, were prepared as described previously [42]. For the effector plasmid, *AtMY75* and *PqMYB4* cDNA fragments were inserted into the *EcoR* I site of p35S-GAL4-DB plasmids. The preparation and transformation of Arabidopsis protoplasts were carried according to the method described previously [43]. The effector, reporter, and internal plasmids were delivered into Arabidopsis protoplast by PEG-mediated transformation of protoplasts, and relative luciferase (LUC/REN) activity was assayed with the Dual-Luciferase Reporter^®^ Assay System (Promega, Madison, WI, USA), using Promega GloMax 20/20 Microplate luminometer (Promega, Madison, WI, USA).

### 4.7. Overexpression Vector Construct and Stable Transformation

For ectopic expression of *PqMYB4* in *A. thaliana*, the full-length cDNA of *PqMYB4* was subcloned from the pMD19-T vector digested with *Kpn* I and *Sal* I to the modified binary vector pCAMBIA1300, digested with *Kpn* I and *Sal* I, generating the pCAMBIA1300-*PqMYB4* overexpression construct. Thus, *PqMYB4* was expressed under the control of the CaMV 35S promoter. This overexpression construct was introduced into *Agrobacterium tumefaciens* strain GV3101 for Arabidopsis transformation. The generated overexpression construct pCAMBIA1300-*PqMYB4* in *Agrobacterium* strain GV3101 was transformed into a wild-type Arabidopsis plant using the floral dip method, as previously described [44]. An *A. tumefaciens* infection solution (OD_600_ = 1.0 − 1.8), containing 5% sucrose and 0.02% Silwet L-77, was prepared to infect inflorescences, and the infection time per inflorescence was 2 min. Subsequently, these plants were transferred to a dark treatment for 24 h. These steps were repeated twice according to the growth state of the plant. The harvested seeds were planted on 1/2 MS plates containing 20 mg·L^−1^ hygromycin in growth chamber at 22 ± 2 °C, under a 15/9 h light/dark (120 µmol·m^−2^·s^−1^) cycle. Hygromycin resistant seedling with green leaves and well-established roots were selected as transformants, and then transferred from the plates to moistened potting soil. The positive transformants was confirmed via the PCR method. The transgenic plants were used for further analysis, and the wild-type non-transformed lines grown in the same conditions.

### 4.8. Total Anthocyanin Content Measurement

The total anthocyanin concentration in tree peony leaves was determined according to the method described previously [45]. Moreover, 0.05 g leaf samples were ground in liquid nitrogen, anthocyanins were extracted with 1% HCL methanol solution for 24 h at 4 °C, then suspended by ultrasound for 60 min. After centrifugation at 12,000 rpm for 10 min, the supernatant was filtered using a 0.22 µm membrane filter and measured at 530 nm and 657 nm by spectrophotometer for absorbance determination. Anthocyanin content was calculated according to the equation ((A_530_ − 0.25 × A_657_) × FW^−1^). Three replicates were analyzed for each sample.

### 4.9. Quantitative Real Time PCR Assay

Quantitative real time PCR (qRT-PCR) assay was carried out to determine the expression level of structure genes and *PqMYB4* gene at different leaf stages in tree peony and the anthocyanin biosynthetic pathway genes in transgenic Arabidopsis. The sequence of anthocyanin biosynthesis related genes in Arabidopsis was obtained from TAIR (https://www.arabidopsis.org/). According to the sequence of *PqMYB4* and related structure genes, qRT-PCR specific primers were designed (Table 1). The qRT-PCR experiments were set up using SYBR Premix Ex Taq II (Takara, Otsu, Shiga, Japan) on StepOnePlus Real Time PCR system (ThermoFisher Scientific, Waltham, MA, USA), following the manufacturer’s recommendation. The reaction mixture (20 μL total volume) contained 10 μL of SYBR^®^ Premix Ex Taq™ II, 0.8 μL of each primer (10 μM), 0.8 μL of diluted cDNA, 0.4 μL of ROX and 7.2 μL of ddH_2_O. The PCR program was carried out with an initial step of 95 °C for 30 s, and 40 cycles of 95 °C for 5 s, 55 °C for 30 s, 72 °C for 30 s; then 95 °C for 15 s, 60 °C for 1 min and 95 °C for 15 s for the dissociation stage. *Pqubiquitin* and *Atactin* were used as reference genes to normalize the expression data [45]. The melting curve program was included at the end of qRT-PCR program, to ensure the specific amplification. The relative expression levels of genes were calculated by the 2^−^^ΔΔ^^CT^ comparative threshold cycle (Ct) method. Three biological replicates were performed for each gene.

## 5. Conclusions

*PqMYB4*, the R2R3-MYB transcription factor gene, was isolated from *P. qiui*, had a C1 conserved motif, an EAR inhibitory motif and a TLLLFR inhibitory motif at the C-terminus. *PqMYB4* expression negatively correlates with anthocyanin biosynthetic gene expression and anthocyanin accumulation in *P. qiui*. Moreover, it was dominantly expressed in green tissues. PqMYB4 has the function of inhibiting the synthesis of flavonoids (anthocyanins).

## Figures and Tables

**Table 1:** Gene specific primers used for qRT-PCR analysis and gene isolation.

Primer Name	Primer Sequences	Usage
PqMYB4-F	AAAGGTACCTACTGGTGTTAGAGAGATTGG	Gene isolation
PqMYB4-R	AAAGTCGACGGTATATTTGGCGATGGATGG	
Pqubiquitin-F	GACCTATACCAAGCCGAAG	qRT-PCR
Pqubiquitin-R	CGTTCCAGCACCACAATC	
PqMYB4-F	TTGACCCTAATAACCATCG	qRT-PCR
PqMYB4-R	TCAAGATTCAAGTCAAGCGAG	
PqCHS-F	CTGCTATCATCATTGGTTCG	qRT-PCR
PqCHS-R	GTCGCTTGTAGTTTTTCGGGT	
PqCHI-F	CTATTCTTTTCACACAGAC	qRT-PCR
PqCHI-R	TGCTTCCCTATGATCGACTCC	
PqF3H-F	CGAAATCCCAATCATCTCC	qRT-PCR
PqF3H-R	TATTTCACGCCAATCTCGCAC	
PqF3′H-F	TGATGTTGATGGAGAGGGT	qRT-PCR
PqF3′H-R	GCTAGGATTTTAGGGTGTCGG	
PqDFR-F	GAGAATATGAGGAAGGTG	qRT-PCR
PqDFR-R	ACACGAAATACATCCATCCAG	
PqANS-F	ACCAGCATCACCAACATCT	qRT-PCR
PqANS-R	GCATATTTCTCCTTCTCTTCC	
Atactin-F	GGAACTGGAATGGTGAAGGCTG	qRT-PCR
Atactin-R	CGATTGGATACTTCAGAGTGAGGA	
AtCHS-F	GCATCTTGGCTATTGGCACTG	qRT-PCR
AtCHS-R	CGTTTCCGAATTGTCGACTTGT	
AtCHI-F	CTCCTCCAATCCATTATTCCTCG	qRT-PCR
AtCHI-R	TTTCCCTTCCACTTGACAGATAGAG	
AtF3H-F	GTGTTTAGCGACGAAATCCCG	qRT-PCR
AtF3H-R	ACGAGCGAGACGAGTCATATCC	
AtF3’H-F	TCGTGGTCGCCGCTTCTAA	qRT-PCR
AtF3’H-R	CCATCGGTGTCCGTAAGGTG	
AtDFR-F	CAAACGCCAAGACGCTACTCA	qRT-PCR
AtDFR-R	CATTCACTGTCGGCTTTATCACTTC	
AtANS-F	ACGGTCCTCAAGTTCCCACAA	qRT-PCR
AtANS-R	CAGCTCCTCAATACAATTCTCACG	
AtUFGT-F	TCGAAGCTACTAAGAATGGTG	qRT-PCR
AtUFGT-R	GGTAACTCGAAAACGGACTTG	
